# Heterotopic Ossification around the Elbow Revisited

**DOI:** 10.3390/life13122358

**Published:** 2023-12-18

**Authors:** Aristeidis-Panagiotis Kontokostopoulos, Ioannis Gkiatas, George I. Vasileiadis, Dimitrios Flevas, Spyridon E. Tsirigkakis, Dimitrios Kosmas, Ioannis Kostas-Agnantis, Emilios Pakos, Ioannis Gelalis, Anastasios Korompilias

**Affiliations:** 1 Department of Orthopaedic Surgery, School of Medicine, University of Ioannina, 451 10 Ioannina, Greece; john.gkiatas@gmail.com (I.G.); tsirigas1989@gmail.com (S.E.T.); kosm.dmt@gmail.com (D.K.); ioanniskostas@hotmail.com (I.K.-A.); epakos@yahoo.gr (E.P.); idgelalis@gmail.com (I.G.); koroban1960@gmail.com (A.K.); 2 Department of Physical Medicine and Rehabilitation, School of Medicine, University Hospital of Ioannina, 451 10 Ioannina, Greece; givasileiadis@yahoo.co.uk; 3 Arthroscopy & Orthopaedic Surgery Department, Metropolitan Hospital, Neo Faliro, 185 47 Pireas, Greece; dflevas@gmail.com

**Keywords:** orthopaedic surgery, trauma, heterotopic ossification, elbow

## Abstract

Heterotopic ossification (HO) is the process of ectopic bone formation in the periarticular soft tissues and is usually formed in the elbow, hip and knee joint as a complication of trauma, burns, brain injury or surgical procedures. The development of HO around the elbow joint can cause a severe limitation of range of motion (ROM) and may affect daily activities of the patient. Treatment of ectopic bone formation around the elbow is a challenge for many surgeons. Non-operative treatment usually fails to restore the ROM of the elbow joint; thus, surgery is necessary to restore the function of the joint. In the past, many surgeons suggested that a delayed excision of HO, until maturation of the ectopic bone, is the best option in order to avoid any possible recurrence. However, many authors now suggest that this delay may lead to complications such as muscular atrophy and formation of soft tissue contractures that can cause a greater impairment of elbow function; thus, early excision is a better option and can better restore the elbow ROM. We performed a literature research of articles that investigated which is the best time of HO excision and we also evaluated if the tethering effect of HO can lead to a greater impairment of the elbow function. We found numerous studies suggesting that a limitation in ROM of the elbow can appear from the tethering of the ectopic bone formation and not only from primary HO. Concerning the HO excision, there were no significant differences between patients who underwent delayed and early excision, concerning the recurrence rate of HO around the elbow. Patients who underwent early excision had better restoration of elbow ROM; thus, early excision, combined with a rehabilitation program, is reported to be the best option for these patients.

## 1. Introduction

Heterotopic ossification (HO) is the process of ectopic bone formation in the periarticular soft tissues. It is a frequent complication of trauma, burns, brain injury or surgical procedures [1,2,3]. Hip, knee and elbow are the most frequently involved joints [1,2].

Ectopic bone on the elbow can affect not only the joint itself but also the muscles around it, as well as the neurovascular structures of the area, thus causing extensive pain. Compression of the overlying skin can also be the cause of this pain. [1,2,3] The development of HO around the elbow may also lead to functional impairment and restricted range of motion (ROM), which can limit Activities of Daily Living (ADL) [4]. The formation of ectopic bone can cause a severe limitation of arc of motion, leaving a ROM of extension less than 100° (from 30° to 130°) [1]. If non-operative treatment fails to restore the ROM of the elbow, surgery becomes necessary to restore elbow function [5].

Many authors suggested in the past that the right time for HO excision is at least one year after the initial injury until the maturation of ectopic bone occurs [5]. Thus, recurrence of HO could be avoided. However, this delay may lead to the formation of soft tissue contractures and the occurrence of muscular atrophy. These complications, in turn, may lead to a greater impairment of elbow function [5]. For this reason, many authors now suggest that the early excision of HO can lead to a better restoration of ROM [5].

This study is a narrative review of the literature and its purpose is to, first, investigate how HO formation affects the ROM of the elbow joint, and second, to assess which is the best time for HO excision and whether the early excision of ectopic bone can better restore the ROM of the elbow. Thus, we can give surgeons a better insight as to which is the best plan to treat patients with ectopic bone formation of the elbow and what to expect depending on the time of HO excision that they choose to perform. An additional objective of this study is to search the current literature and investigate whether the tethering effect of HO can lead to a reduction of ROM.

## 2. Material and Methods

We performed a computer-based literature search of articles, assessing the influence of HO formation to the ROM of the elbow joint and investigated when is the best time for ectopic bone excision. We identified all relevant articles through systematic research on two online databases, PubMed and Cochrane Library. The keywords used for this research included “heterotopic ossification”, “range of motion” or “ROM”, and “elbow”. We also performed manual research of the reference lists of all the included articles.

## 3. Pathophysiology

In HO, the formation of ectopic bone is believed to be a result of the inappropriate differentiation, migration and proliferation of pluripotential mesenchymal cells into osteoblastic stem cells [6,7]. In trauma cases, products of the torn muscle, torn soft tissue and bleeding can influence this process [7]. Urist et al. suggested that there is a hormone-related mechanism that leads to this differentiation. Bone morphologic protein (BMP) has been identified as the possible protein that determines whether a pluripotential mesenchymal cell differentiates to form bone or cartilage instead of muscle or scar [8,9].

Systemic factors have been identified to play a key role in ectopic bone formation in patients with traumatic brain injuries (TBI). Many patients have developed HO in the elbow despite having no traumatic injuries to this joint [6,7]. BMP and Prostaglandin-E2 have been found to play a role in the development of ectopic bone in patients with Central Nervous System (CNS) injury as well [6,7].

The affected tissues in burn injuries cause inflammation of the site [10]. The inflammatory signals such as BMP, tumor necrosis factor and interleukin-6 secretion cause the increased osteogenic differentiation of the mesenchymal cells, resulting in ectopic bone formation [10].

There are differences between the formation of normal and ectopic bone [7]. The periosteum which envelopes the normal bone, does not cover the external surface of the ectopic bone [7]. In ectopic bone there are three zones that can be identified histopathologically. The outer zone consists of highly organized bone tissue [7]. The inner zone consists of dense cells surrounded by osteoid, which is the medial zone [7]. The amount of osteoblasts and osteoclasts is higher in ectopic bone in comparison to the normal bone [7].

## 4. Risk Factors

Ectopic bone formation occurs under numerous circumstances; thus, it is difficult to define the specific cause of HO. It is even more challenging when the patient suffers from more than one condition, like elbow trauma, traumatic brain injury or burns. Under these circumstances it is difficult to directly associate HO with one specific condition. On the other hand, there is an add-on phenomenon that increases the risk of HO appearance [11].

Direct elbow trauma is the main cause of ectopic bone formation on the elbow joint [7]. The extent of HO formation depends directly to the severity of the injury [7,12,13]. Fractures and dislocations of the elbow can cause an increased incident of HO, while terrible triad injuries can cause a five times higher chance of HO development [7,12,13,14]. Following elbow fractures, the prevalence of HO is approximately 40%, while a delay in intervention increases the risk of ectopic bone formation [13,15,16].

Spinal cord and traumatic brain injuries can increase the prevalence of HO [14]. The relationship between neurogenic heterotopic ossification (NHO) and the central nervous system is not completely understood, but it appears to be related with peripheral neurotransmitters and inflammatory mechanisms that affect osteoblast formation [17,18] The prevalence of NHO in patients with spinal cord injuries can be affected by the severity of the injury and the level of the spinal cord injury, as thoracic and cervical spine injuries lead to more severe NHO [18]. Additional factors such as severe spasticity, impaired cognition, urinary tract infections, deep venous thrombosis and/or tracheostomy increase the risk of NHO [7,18]. The risk factors in patients with traumatic brain injury are similar to them in spinal cord injuries [14]. A major difference between these two injury mechanisms is that, in spinal cord injuries, the ectopic bone usually forms at a level lower than the level of the neurological injury to the injury level and most commonly at the hip, while patients with traumatic brain injury may develop HO throughout the body, including the elbow joint [14].

Among patients with burn injuries, the development of HO is more related to the degree of thermal injury and the percentage of body surface area affected [19,20]. Burns requiring skin grafts and burns covering more than 30% of the total body surface area are the most important risk factors [20]. An additional risk factor is the development of third-degree burns at or near the elbow joint [19]. Scar contractures from HO near the elbow joint caused by the burn injury can be formed, thus affecting the ROM of the joint [14]. The elbow joint is the most commonly affected joint among patients with thermal injuries [14]. However, there are numerous factors that may cause the development of HO in these patients [7]. Prolonged immobility, elbow injury with concurrent paralysis and a possible undiagnosed elbow injury in these patients are some of the factors that may lead to ectopic bone formation [7]. Thus, it is challenging to define the specific cause of HO occurrence [7].

## 5. Tethering Effect of HO

The development of HO on the elbow joint can lead to functional impairment and restricted ROM. A limitation in ROM can appear not only from primary HO, but also from a variety of other causes that may be relevant to the primary HO development [21,22]. The factors that affect elbow motion are divided into blocks to motion and tethering constraints [22]. Both blocks to motion and tethering constraints may lead to extension dysfunction and can cause loss of elbow flexion or/and loss of pronation/supination as well [21,22,23]. Blocks to motion that can appear both anterior (osteophytes, HO, malunion) and posterior (loose bodies in the olecranon fossa, olecranon osteophytes) can lead to elbow dysfunction [21,22]. A tether, causing limitation in extension and flexion of the elbow, can also appear both anterior and posterior [21,22]. However, tethering caused specifically by HO development mostly appears anterior and is usually the result of HO of the anterior band of the medial collateral ligament [22]. The functional impairment in these occasions is not cause by HO per se, but from the tethering effect, which originates from the primary ectopic bone formation [21,22].

Tan et al. studied the outcome of elbow release on 52 patients, which entailed removing the tethers that caused limitation on elbow ROM [21]. Some of the patients undergoing this surgery, suffered from HO [21]. After excising these tethers, the target for each patient was to regain ROM of the elbow by manipulating the joint in flexion and extension or pronation and supination [21]. This approach had great success in the majority of the patients, as the authors observed improvements in elbow ROM and 83% of the patients regained functional motion after removing the tethers [21]. However, in this study it was reported that a relatively high rate of patients (38%) required subsequent procedures in order to eventually reach a satisfying result, concerning the function of the elbow joint [21].

Sun et al. also conducted a study where they performed open arthrolysis on 49 patients with severe loss of motion as a result of trauma [22]. Simultaneously with the arthrolysis they performed the excision of tethers from these patients [22]. Postoperatively, all patients underwent rehabilitation consisted of three different stages in order to regain the function of the elbow joint [22]. In this study, authors observed a higher rate of success on patients, as 98% of the regained functional ROM after surgery [22]. A total of 18% of these patients developed complications but none of them underwent any additional procedure for any of these complications, following the first surgery [22].

Vasileiadis et al. in 2015 published an article where they highlight the tethering effect caused by HO formation. In their study, 47% of patients have restricted the motion under investigation (forearm rotation) for reasons unrelated to HO, i.e., post traumatic fibrous adhesions or/and soft tissue contractures after inflammation and prolonged immobilization [23].

## 6. Prophylaxis for HO

Many authors have investigated the possible effect of Non-Steroidal Anti-Inflammatory Drugs (NSAIDs) and radiotherapy in the prevention of HO of the elbow joint after a surgical procedure [24,25,26,27,28,29]. There are studies supporting NSAIDs for the prevention of elbow HO, while other studies suggest that NSAIDs are not efficacious for the prophylaxis of elbow HO [24,25]. Antonacci et al. suggested that prophylaxis with NSAIDs (75 mg/d of indomethacin for 3 to 4 weeks) appears to reduce the incidence of HO, while Bochat et al. reported that NSAIDs (25 mg/d of celecoxib, ibuprofen, indomethacin, meloxicam, or naproxen from 3 days to 6 weeks) did not affect the incidence of HO in patients who underwent elbow surgery for trauma [24,25]. Atwan et al. also concluded that there was no significant difference in the incident of HO between a group that received indomethacin after elbow trauma and a control group [30]. On the other hand, Mishra et al. and Geller et al. proved that the use of radiotherapy (6-MV photons with a single fraction of 7 Gy, 24 h preoperatively or 48 h postoperatively) for the prevention of HO can be safe and efficacious, and was not shown to be associated with sarcoma caused by radiation [26,27]. Mohamed et al. and Lee et al. concluded that postoperative radiotherapy is efficacious and safe and any possible treatment failure is mainly a result of surgical difficulties due to advanced HO on the joint [11,31]. However, there is lack of evidence supporting the use of radiotherapy on burn patients because of the possible effects on soft tissues, especially on patients with skin grafts [32]. An interesting finding by Strauss et al. is that radiation prophylaxis for HO can be safe and effective, but it can even be more efficacious in combination with NSAIDs (6-MV photons with a single fraction of 5 to 7 Gy within the first day postoperatively combined with 150 mg/d of indomethacin for 10 days) [28]. Henstenburg et al. performed a systematic review comparing options for HO prophylaxis after elbow trauma [29]. The authors reviewed a total of 36 articles and 826 elbows [29]. Nearly 25% of them received radiation therapy and 75% of them received NSAIDs in a wide variety of dosage and duration [29]. The authors found no statistical difference in HO development or recurrence between these two groups, thus suggesting that no option is superior in terms of efficacy and surgeons should choose the prophylaxis option based mainly on patient characteristics [29]. The role of kinesiotherapy in the prevention of HO is also being studied. Although researchers recommend controlled passive range of motion exercises, there are not enough high quality studies yet that can support the conclusion that kinesiotherapy is effective in the prevention of ectopic bone formation on the elbow joint [33].

## 7. Treatment of HO

Conservative treatment is indicated for patients that have elbow stiffness due to HO for less than six months [33]. Current non-surgical treatment measures include physical therapy and manipulation under anesthesia in order to restore the ROM of the involved joint [34]. However, these options have limited effects in HO treatment and can be used mainly in HO that causes a small limitation of ROM [34]. Surgical options are necessary if nonoperative management fails to restore the elbow function and ROM after 6 months [1,28]. Surgical excision of ectopic bone and contracture release, if present, are the most common surgical options in elbow HO management [34].

However, the main issue concerning the treatment of elbow HO is to specify the appropriate excision time of the ectopic bone. In the past years, many authors suggested that delayed surgical excision of HO, 12 to 24 months from injury to operation is optimal [35,36,37,38,39,40]. In this period, the maturation of ectopic bone occurs and there is enough radiographic evidence of that maturation [35,37]. Waiting for the ectopic bone to mature and then performing the surgical excision reduces the incidence of recurrence [35,36,37,38,39]. Nevertheless, this delay usually leads to further impairment of the elbow function as, in this period, the intensity of pain is increased, muscular atrophy occurs and secondary contractures are formed [40,41,42]. Thus, many authors studied the possibility and outcome of early HO excision and many suggested that this approach is more beneficial for the patients with ectopic bone formation on the elbow [1,5,32,43]. These findings are in accordance with the results of a systematic review of the literature by Almangour et al. to determine whether, in patients with neurological HO, the timing of surgery affects the risk of HO recurrence [44]. They imply that early excision of the HO should allow the patient to reach their functional potential because they will not be impeded by a restriction in movement [44].

Chen et al. reviewed 164 patients who underwent HO excision after a post-traumatic stiff elbow [1] (Table 1). These patients had grade IIA, IIC, IIIA or IIIC HO, based on the Hastings classification system [1]. Fifty-two of these patients underwent early excision of ectopic bone at an average of 6 months, while the control group, which was comprised of 112 patients, underwent delayed excision of HO at an average of 23 months [1]. Postoperatively, all patients participated in physical therapy and rehabilitation [1]. Recurrence incidence of HO postoperatively was the same in both groups, as recurrence was found in 15 of 52 patients (28.9%) within the early excision group, and in 30 of 112 patients (26.8%) within the control group [1]. Further surgical procedures were performed only in patients that were not satisfied with the function of their elbow and in patients with ankylosis in the pronation–supination plane [1]. Improvement on ROM, regarding flexion, extension, pronation and supination, was similar in the early excision group and the control group [1]. The postoperative Mayo Elbow Performance Score (MEPS) was the same on both groups [1]. Thus, the authors suggested that delaying the excision of HO has no benefits concerning the recurrence rates and early excision associated with a rehabilitation protocol can lead to decreased recurrence incident and better function of the elbow joint [1].

In another study, He et al. reviewed 42 patients with HO, who underwent surgical excision [5] (Table 1). In the early excision group, 17 patients underwent excision at an average time of 7 months, and in the late excision group (control group), 25 patients underwent excision at an average time of 33 months [5]. Both groups had similar ROM and MEPS preoperatively [5]. Physical therapy was performed on all patients after the surgery, which mainly consisted of exercises for flexion and extension [5]. The early excision group and control group showed significant improvements of ROM and MEPS [5]. However, early excision of HO statistically improved ROM and MEPS more than late excision [5]. Furthermore, both groups had a similar recurrence incident [5]. Within the early excision group, recurrence was found in 4 of 17 patients (23%), and within the late excision group, recurrence was found in 6 of 25 patients (24%) [5]. Therefore, not only did the authors suggest that late excision has no advantage regarding the recurrence incident, but they also found that early excision can lead to greater restoration of elbow ROM and function [3].

In the studies by Chen et al. and He et al., authors compared the early excision of ectopic bone versus the late excision of HO [1,5]. Numerous studies evaluated only the early excision in small groups of patients with HO of the elbow. (Table 2) Moritomo et al. performed an early excision of ectopic bone on nine patients with HO of the elbow [42]. These patients underwent surgery at an average time of 8 months [42]. Postoperatively, all patients had improved ROM and function of the elbow and had no symptoms of instability or pain [42]. A higher recurrence rate was observed in this group of patients, as five of nine patients (55%) had spotty HO after the surgery [42]. However, the small number of patients does not allow us to correctly evaluate the recurrence rate on this study, and the authors also noted that all patients with spotty HO postoperatively had a sufficient ROM of the elbow [42]. Yang et al. also evaluated a small group of seven patients with HO of the elbow [43]. The patients underwent early excision of HO at an average time of six months since initial injury [43]. Recurrence rate on these patients postoperatively is not provided by the authors [43]. However, the functional recovery of the elbow on these patients is interesting [43]. Six of the seven patients had nearly full recovery on function and ROM of the elbow joint after the excision of HO [43]. Only one patient had insufficient ROM after the surgery, which was not easy in daily life [43]. This patient underwent HO excision 12 months after initial injury, while all the other patients underwent excision only until 7 months after initial injury [43]. Thus, the authors suggested that delayed excision time of ectopic bone may be one of the reasons for functional impairment after the surgery [43].

Chen et al. supported the early excision of HO, combined with immediate postoperative physical therapy [32]. They reported three cases, patients with burn injuries who suffered from HO on elbow joint [32]. All patients had unsatisfactory ROM during their hospitalization for their burn injuries and all patients underwent surgery as soon as it was possible, due to their additional medical problems associated with the primary injury [32]. The first patient underwent surgery 12 months postburn, the second patient underwent surgery 10 months postburn and the third patient underwent surgery 6 months postburn [32]. All patients were reported to have great recovery regarding ROM of the elbow joint and no recurrence of HO was noted postoperatively [32]. Malca et al. also conducted a study regarding postburn HO [45]. The authors reported in their study that 35 patients had HO related with burn injuries and these patients underwent HO excision an average time of 10 months after the initial burn [45]. Only two patients were reported to have a recurrence of HO after the surgery [45]. Tsionos et al. studied 35 elbows (28 patients) with HO related to burn injuries [40]. The mean time between the initial burn and the ectopic bone excision was 12 months [40]. It is interesting that the authors reported 25 elbows (71%) with presence of ossified periarticular tissue on radiographs postoperatively [40]. However, from these elbows only four (11%) were considered as recurrences of HO, as only these four elbows showed further restriction of elbow movement [40].

In every paper on Table 1 and Table 2, the surgeons performed clinical examination to diagnose the ectopic bone formation on the elbow joint [1,3,27,34,36,37,39]. The main criteria for the diagnosis was a reduced ROM (less than 100°), which affected the daily routine of their patients [1,3,27,34,36,37,39]. Subsequently, they performed preoperative X-rays and confirmed the ectopic bone formation on the elbow joint during the surgery [1,3,27,34,36,37,39]. The patients in all those papers were adults (over 18 years old) and no significant difference was observed concerning the recurrence rate of HO or the optimal time of HO excision, in terms of age [1,3,27,34,36,37,39].

## 8. Rehabilitation

Postoperative management of patients with HO is of great importance in order to achieve a functional ROM and to avoid any possible HO recurrence [1,5,46]. Usually, a long arm splint is being applied postoperatively on patients who underwent surgical excision of ectopic bone on the elbow joint [46]. A unilateral hinged external fixator can be applied on patients who underwent HO excision for 4 weeks [5]. On the first or second postoperative day, the patient begins physical therapy consisted of active assisted and mild passive flexion and extension exercises [1,5,47]. Flexion and extension exercises continue until the ROM is no longer changed by these exercises [5]. During the first week postoperatively, exercises can be performed for half an hour three times a day [1]. After the first week the length of these exercises can be extended to 1 h [1]. Exercises are individualized for each patient and all patients are encouraged to participate at the rehabilitation program for 4 to 6 months [1,5]. NSAIDs have been proved to be efficient for the prevention of ectopic bone formation [48]. Thus, many authors suggest the administration of indomethacin for 4 weeks at a dose of 25 mg three times a day to prevent a possible HO recurrence [1,5,32,47]. The use of radiotherapy for the prevention of HO recurrence has been studied numerously in the past [49,50]. There are studies that support the effectiveness of radiotherapy on the prevention of HO, instead of NSAIDs [49]. Surgeons usually prefer the use of NSAIDs because of the possible risks of radiation on operational site [32]. However, Geller et al. proved that radiotherapy for the prophylaxis for HO recurrence can be safe and is not related with sarcoma cause by radiation [27]. A major problem is that there is lack of evidence supporting the use of radiotherapy in burn patients, as there are risks and possible soft tissue complications, especially on patients with skin grafts [32]. A systematic review article conducted by Vasileiadis et al. on the role of kinesiotherapy in the prevention of HO formation concluded that firm conclusions cannot be drawn about the effectiveness of kinesiotherapy. Nevertheless, it is recommended that controlled passive ROM (PROM) exercises (especially CPM) be applied early and pain-free, especially in the neurogenic HO patients, while active ROM in painless limits is beneficial in the HO prevention of traumatic elbows or burn joints [33].

## 9. Conclusions

Heterotopic ossification is the formation of ectopic bone in the periarticular tissues following trauma, burns, brain injury or surgical procedures. Symptoms of HO of the elbow joint are pain, functional impairment and restricted ROM. There are numerous risk factors that can lead to HO of the elbow, and it can be difficult to directly associate HO with one of these risk factors. On the other hand, there is an add-on phenomenon that increases the risk if multiple risk factors are present. Nonsteroidal anti-inflammatory drugs and radiotherapy are two medical treatments that are widely used for prophylaxis against HO development in the elbow joint and they both seem to be safe and effective in most of the patients. It is suggested that, when administered in combination, they can be even more effective to prevent ectopic bone formation. In general, the role of kinesiotherapy in the prevention of HO formation has not been clarified yet. On the other hand, kinesiotherapy with entire ROM in painless limit is indicated in order to prevent HO formation around the elbow.

For mature HO that causes functional impairment and ROM limitation, surgical treatment is necessary. Surgeons perform ectopic bone excision in patients with HO in the elbow joint. One of the greatest dilemmas is what is the right time to perform this surgical excision. Early surgery for patients with HO of the elbow leads to a sufficient ROM postoperatively, and does not cause an increased recurrence rate compared to the patients who underwent delayed surgery. In addition, early surgery does not lead to the possible secondary effects that the delayed excision may have like greater functional impairment of the elbow, increased pain, muscular atrophy and secondary contractures. Thus, early excision of ectopic bone associated with a rehabilitation protocol is reported to be the best option for patients with HO of the elbow.

## Figures and Tables

**Table 1:** Studies that compared early and late excision of HO.

Study	Patients	Early Excision (Average Time)	Recurrence Rate, Early Group	Late Excision (Average Time)	Recurrence Rate, Late Group
Chen et al. [1] (2015)	164	52 patients (6 months)	15/52 (28.9%)	112 patients (23 months)	30/112 (26.8%)
He et al. [3] (2018)	42	17 patients (7 months)	4/17 (23%)	25 patients (33 months)	6/25 (24%)

**Table 2:** Studies that evaluated only early excision of HO.

Study	Patients	Excision Time	Recurrence Rate
Moritomo et al. [36] (2001)	9	8 months	5/9 (55%)
Yang et al. [37] (2002)	7	6 months	Not provided (insufficient ROM for the patient the longer excision time)
Chen et al. [27] (2019)	3 (case reports)	9 months	No recurrence
Malca et al. [39] (2018)	35	10 months	2/35 (5%)
Tsionos et al. [34] (2004)	28 (35 elbows)	12 months	4/35 (11%)

## Data Availability

Data are contained within the article.
